# Assembly and Maintenance of Sarcomere Thin Filaments and Associated Diseases

**DOI:** 10.3390/ijms21020542

**Published:** 2020-01-15

**Authors:** Kendal Prill, John F. Dawson

**Affiliations:** Centre for Cardiovascular Investigations, Department of Molecular and Cellular Biology, University of Guelph, Guelph, ON N1G 2W1, Canada; kprill@uoguelph.ca

**Keywords:** sarcomere assembly, sarcomere maintenance, sarcomere thin filaments, thin filament assembly, thin filament maintenance, thin filament turnover, actin turnover, chaperone, sarcomere, myopathy

## Abstract

Sarcomere assembly and maintenance are essential physiological processes required for cardiac and skeletal muscle function and organism mobility. Over decades of research, components of the sarcomere and factors involved in the formation and maintenance of this contractile unit have been identified. Although we have a general understanding of sarcomere assembly and maintenance, much less is known about the development of the thin filaments and associated factors within the sarcomere. In the last decade, advancements in medical intervention and genome sequencing have uncovered patients with novel mutations in sarcomere thin filaments. Pairing this sequencing with reverse genetics and the ability to generate patient avatars in model organisms has begun to deepen our understanding of sarcomere thin filament development. In this review, we provide a summary of recent findings regarding sarcomere assembly, maintenance, and disease with respect to thin filaments, building on the previous knowledge in the field. We highlight debated and unknown areas within these processes to clearly define open research questions.

## 1. Introduction

Striated muscle requires the coordination of hundreds of proteins not only for cellular function but also for assembly of the contractile sarcomere units within the myofibril.

While the assembly and maintenance of myosin thick filaments and titin within the sarcomere have been the spotlight of many studies in muscle development and myopathies, the formation and maintenance of the thin filaments have been largely under-characterized despite the suite of myopathies originating from components of thin filament development. The purpose of this review is to present the discoveries of thin filament assembly and maintenance in the last decade. We will combine these findings with previous knowledge of thin filament development to highlight a model for thin filament assembly/maintenance and how this relates to muscle disease. Many factors discussed in this review have alternative names listed in Table 1. Sarcomere thin filament assembly and maintenance is highly conserved across striated muscle with a few isoform and tissue-specific exceptions. We discuss these cardiac and skeletal sarcomere thin filament differences in the appropriate sections below.

## 2. Thin Filament Assembly in the Sarcomere

**Figure 1:**
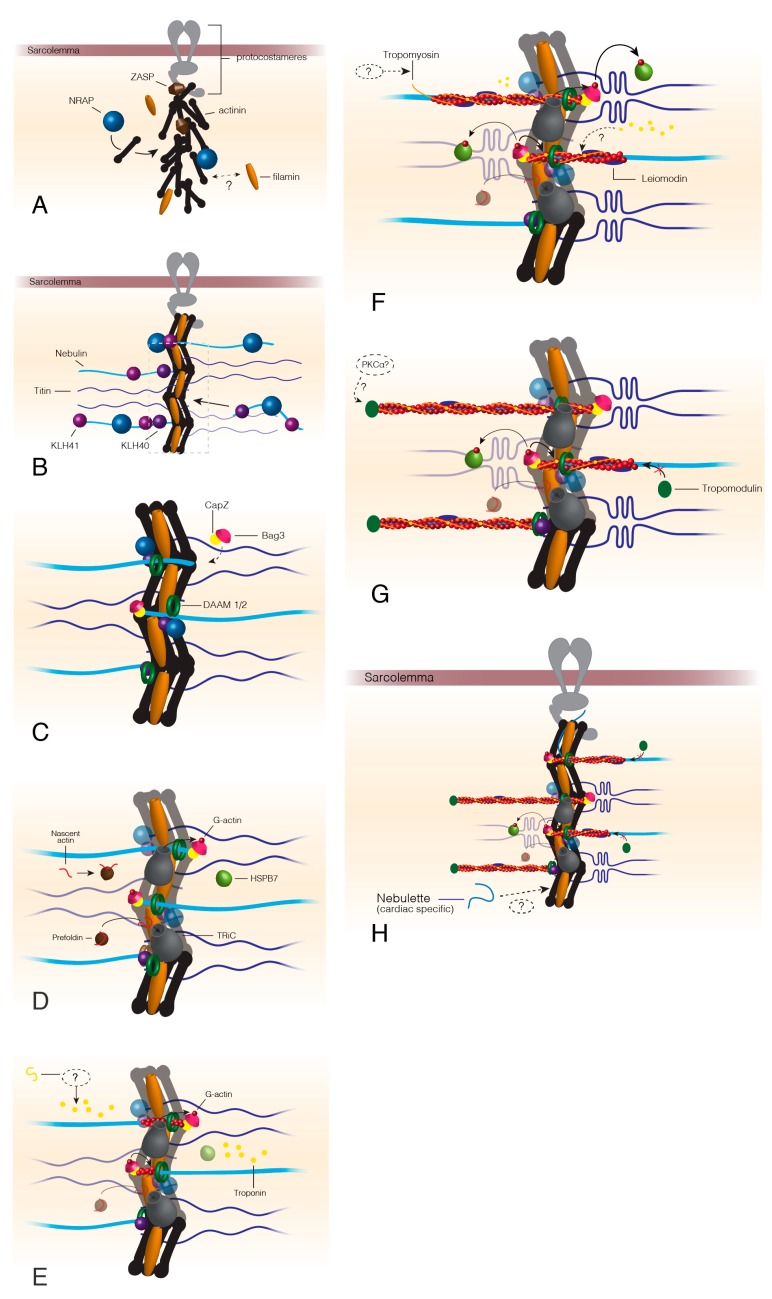
Thin filament assembly beginning at the formation of the Z-disc. The formation of the Z-disc begins once ZASP is recruited to the sarcolemma via proteins of the protocostameres (**A**). ZASP localization draws actinin, the actinin chaperone, NRAP, and filamin C to the protocostameres (**A**). Actinin and filamin C organization allows for nebulin/nebulette and titin incorporation into the premature Z-disc (**B**). KLHL40 and KLHL41 factors stabilize nebulin as it is incorporated into the Z-disc and prevent nebulin aggregation (**B**). Bag3 localizes CapZ to the Z-disc at approximately the same time as DAAM1/2 appears in the Z-disc (**C**). Actin chaperones prefoldin and TRiC localize to the Z-disc after DAAM1/2 and Bag3. Prefoldin targets nascent actin to TRiC for folding into its final conformation. TRiC transfers folded G-actin to Bag3, which provides actin monomers for filament formation by formin proteins (**D**). HSPB7 arrives at the developing thin filaments around this time to regulate the speed of actin polymerization. Troponin localizes to the I-band by an unknown mechanism (**E**), before tropomyosin, which is also incorporated by an unknown method (**F**). Leiomodin is recruited to polymerizing actin thin filaments (**F**) and functions to both stabilize the growing filament but also prevent tropomodulin capping that stops actin polymerization (**G**). Once thin filaments reach their mature length, leiomodin no longer competes with tropomodulin and the thin filaments are capped by tropomodulin possibly following phosphorylation by PKCα (**G**,**H**). Dashed lines indicate unknown or hypothesized mechanisms/proteins.

The site of thin filament formation begins with the development of the Z-disc at the protocostameres (premature costameres or sarcomere-to-membrane attachment) and muscle cell membrane (Figure 1). Protocostameres recruit ZASP, a member of the Alp/Enigma protein family, to the sarcolemma, followed by the ZASP-dependent localization of α-actinin [1,2,3,4]. α-actinin is organized into the characteristic Z-disc lattice by N-RAP, which remains in the sarcomere to act as a scaffold for thin filament assembly within the Z-disc [5,6,7]. Sarcomere filamin localizes early to the developing Z-disc but the reason for this localization is not clear and a filamin chaperone remains to be identified [8]. Filamin acts as a second anchor for thin filament attachment to the Z-disc and aids in linking thin filaments of adjacent sarcomeres together [9]. Although myopodin (SYNPO2) is expressed before α-actinin, a small amount of in vitro work demonstrates myopodin colocalizes with filamin C and α-actin during myofibril maturation suggesting myopodin may require both sarcomere proteins to be properly recruited to the sarcomere [10].

Calsarcin localization coincides with ZASP recruitment to the protocostameres [11]. Calsarcin binds α-actinin, filamin C early in sarcomere formation followed by T-cap at the time of its integration into the Z-disc [12]. Calsarcin is important for regulating calcineurin-mediated signaling by sequestering calcineurin to the Z-disc [13].

Once the nascent Z-disc has formed, the protein giant, nebulin, is incorporated into the Z-line of skeletal muscle [14,15,16]. Low levels of nebulin have been detected in the sarcomere thin filaments of cardiomyocytes [17] but another member of the nebulin family, nebulette, is predominantly expressed in cardiac tissue [18,19,20,21]. For a concise comparison of nebulin family members in striated muscle, see [22]. Nebulette and nebulin are homologous in function and have similar protein structure but far more studies have been conducted on dissecting nebulin and its role in skeletal sarcomere assembly and maintenance, which is discussed in this review [22]. While no chaperones have been identified for nebulin folding, NRAP, KLHL41 (skeletal muscle-specific factor), and KLHL40 bind to nebulin and prevent the giant from aggregating or degrading [23,24,25,26]. The binding of these chaperones may result in the localization of nebulin to the Z-disc. Nebulin acts as a thin filament scaffold, providing structural stability to the sarcomere thin filaments during contraction. Thin filaments can form without nebulin but quickly degrade once sarcomere contractions begin [27,28,29,30]. There are two competing models for nebulin function in sarcomere assembly: The first suggesting that nebulin simply acts as a molecular ruler and determines thin filament length [28,29,31,32], while the second model states that nebulin is only required for thin filament assembly and stability and does not dictate thin filament length [33,34]. This latter model proposes that there are two segments of the actin thin filaments: nebulin-bound and nebulin-free [34]. The nebulin-free segment is regulated and stabilized by other thin filament proteins such as tropomodulin that caps the end of the thin filaments. Thin filament length varies across fiber and tissue type, possibly to accommodate the different force generation demands of each muscle type. While we cannot review nebulette to the same extent, some studies have shown that nebulin and nebulette properties and sarcomere physiology are homologous, suggesting that nebulette may also utilize the same chaperones and proteolysis components [22].

Based on immunostaining of titin as a landmark of myofibril assembly (occurs simultaneously with nebulin), myotilin integrates into the Z-disc at the stage of titin incorporation [35]. Myotilin binds filamin C, F-actin, and α-actinin and acts as an additional anchor/force absorber at the Z-disc [36,37]. Whether myotilin is necessary for sarcomere assembly is debated in the literature [35,38]. CapZ localizes to the Z-disc, with aid from Bag3, before actin is recruited for thin filament polymerization [39,40]. CapZ will bind the C-terminal end of nebulin and helps direct and regulate actin polymerization [41,42]. CapZ is also the site of chaperone and sarcomere maintenance factor localization, acting as a focal point for mechanosensory pathways [40,43,44].

Factors DAAM 1 and 2 appear at the Z-disc before actin filament assembly and recruit the critical factor, profilin, for actin polymerization [45,46,47]. DAAM1/2 mouse knockout models display disrupted cardiac sarcomere assembly and intercalated disc structures [48] and DAAM *Drosophila* mutants demonstrate disorganized sarcomeres with reduced thin filament numbers [49]. Since DAAM1/2 are members of the formin family of proteins, DAAM1/2 possess formin homology domains 1 and 2 (FH1 and FH2, respectively). The FH2 domains of a formin are arranged in a ring-like shape around the growing barbed ends of the actin filament [50,51]. Competitive binding between free profilin and actin-bound profilin to the FH1 domain may regulate the speed of actin thin filament formation [52,53]. It is debated in the literature whether an additional formin, FHOD3, is necessary for sarcomere thin filament assembly [54,55,56] or maintenance alone [45,57,58]. Unlike cytoskeletal actin polymerization, which can be initiated by the Arp2/3 complex in the dendritic model of cell migration [59], recent work found thin filament assembly in muscle sarcomeres does not require Arp2/3 but rather involves FHOD3 [60]. The thin filament maintenance aspect of formins will be discussed in a later section.

It is unclear if the co-chaperone, Bag3, arrives just prior to or simultaneously with the actin chaperones TRiC and prefoldin [40,61]. Prefoldin targets nascent actin to TRiC, which is localized within the Z-disc [61]. TRiC folds and transfers G-actin (actin monomer) to Bag3 that is bound to CapZ. Together, bag3, CapZ, and formin stabilize and begin actin polymerization along the nebulin scaffold [54,56,62,63]. HSPB7 regulates thin filament length by sequestering G-actin from the bag3-CapZ-formin-nebulin complex. In mice, mutants of HSPB7 are embryonic lethal with actin filament aggregates and longer than normal thin filaments [64].

The following steps of thin filament assembly occur simultaneously with actin polymerization. Troponin T localizes to the developing thin filament and is required for the recruitment of tropomyosin [65,66]. Troponin T is part of a transcriptional feedback pathway as abnormal expression of troponin T (overexpression or no expression) results in the down-regulation of troponin T, C, I, actin and tropomyosin [67,68,69]. Troponin T may serve as a checkpoint for proper sarcomere assembly with respect to the I-band region. No chaperones have been identified for the troponin subunits or tropomyosin.

Tropomyosin incorporation recruits leiomodin (lmod) to the thin filament and is required for the addition of tropomodulin [45,70]. Leiomodin binds to the growing ends of the actin filament and helps stabilize G-actin polymerization while inhibiting tropomodulin binding to the ends of the thin filaments [45,71]. Tropomodulin binding or “capping” prevents further actin polymerization and is critical for regulation of thin filament length. The length of thin filaments is dependent on the temporal tradeoff of leiomodin and tropomodulin. Higher concentrations of leiomodin at the thin filament during assembly ensure filament growth with lmod mutants displaying short thin filaments that result in dysfunctional contraction and force generation. As the assembly is completed, leiomodin concentrations must decrease to allow for tropomodulin binding at the ends of the thin filaments [72]. The mechanism behind the tradeoff between leiomodin and tropomodulin is not well established but may include post-translational modifications by protein kinase, PKCα, or targeted protein turnover by calpains or the ubiquitin-proteasome system [73,74].

## 3. Sarcomere Thin Filament Maintenance

### 3.1. Thin Filament Maintenance and Turnover

Sarcomere maintenance can be subdivided into two categories of factors: those that refold/reassemble the sarcomere structure and those factors that remove nonfunctional proteins from the cell. Factors of the former are likely chaperones and structural proteins such as those discussed in the previous section. Many factors involved in the assembly also act as maintenance factors, complicating the identification of activities specific to maintenance. Without conditional knockout studies, many factors are only classified as assembly factors since development does not proceed past sarcomere formation in their absence. Maintenance factors of the second category come from the suite of systems that remove sarcomere proteins that can no longer maintain functional integrity. These two categories of thin filament quality control must work together to properly maintain sarcomere thin filaments throughout the life of the organism.

There are two main processes for thin filament maintenance: 1) autophagy and 2) single protein degradation. Single protein degradation happens through the Ubiquitin-Proteasome System (UPS), following isolation from the myofiber by calpains in most cases. The UPS is one of the major quality control pathways for thin filament maintenance and turnover with many proteins destined for degradation by the proteasome [75]. This section will focus on factors that monitor and target thin filament and thin filament associated proteins for turnover (e.g., Bag3).

### 3.2. Calpain Mediated Breakdown of Z-Disc and Thin Filaments

**Figure 2:**
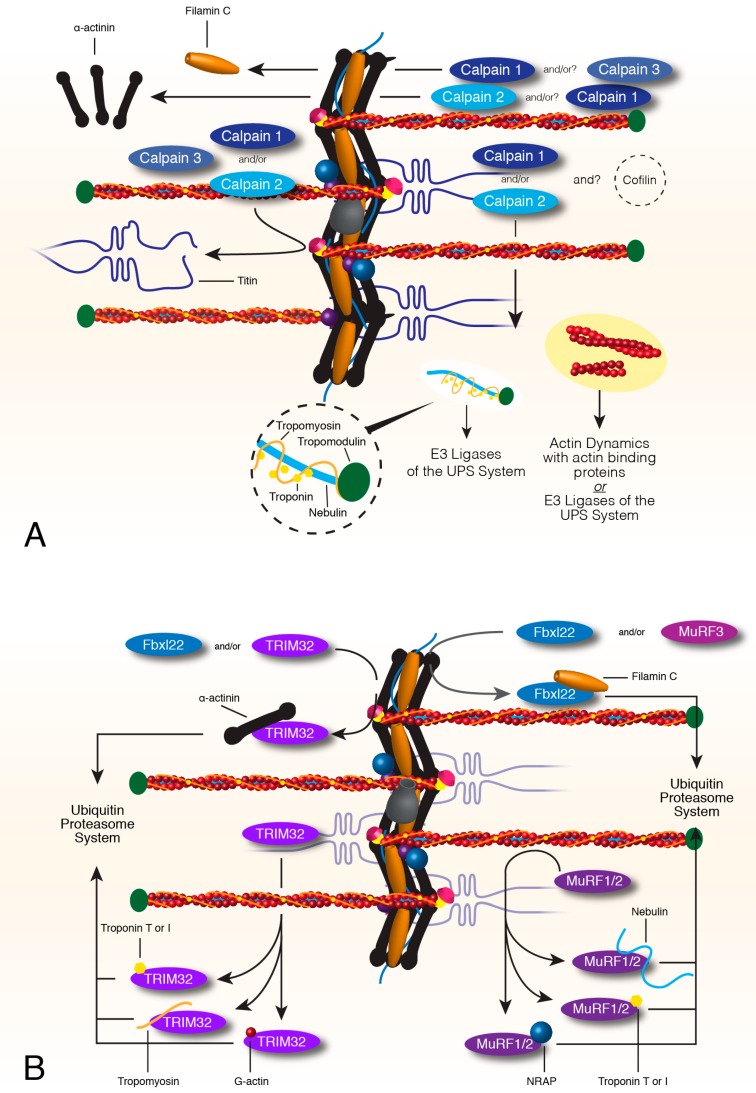
Calpain and Ubiquitin Proteasome Turnover of sarcomere thin filaments and associated factors. Calpains 1, 2 and 3 remove or release proteins from the complex contractile network of the sarcomere. A combination of calpains can release filamin C, actinin, titin, and the sarcomere thin filament, although actin is not degraded by calpains (**A**). Targets of the calpain system that are removed from the sarcomere become target proteins of the UPS system. MuRF1 and 2 can target proteins of the thin filament such as nebulin, troponin T and I and the nebulin chaperone, NRAP, for proteasome degradation (**B**). MuRF3 or Fbxl22, the recognition factor of the SCF complex, target filamin C for degradation. TRIM32 can also target proteins of the thin filament such as troponin T and I, tropomyosin and actin, for degradation. TRIM32 or Fbxl22 can also target actinin for ubiquitin-dependent degradation (**B**). All targets of the E3 ligases are directed to E2 and E1 ligases for ubiquitination and eventually degradation by the proteasome.

Within the last decade, the calpain protease family has emerged as an important class of factors involved in intracellular protein turnover [76]. Unlike other proteases that degrade their substrates, calpains recognize their substrates, but do not degrade them [76]. Although not well understood, calpains can target and release individual or protein complexes from the sarcomere (Figure 2A) [76]. The function of calpains appears to be the first step in a pathway required for sarcomere protein turnover as members of the ubiquitin-proteasome system (UPS) are unable to degrade some sarcomere proteins in the absence of calpains—this could be due to the complexity of the sarcomere and that the UPS is unable to segregate their client proteins [76,77,78]. Among the calpain family, only three have been identified in muscle development and health: calpain 1, 2, and 3 [77]. The calpains share functional redundancy, targeting and segregating many of the same sarcomere proteins, although calpain 1 and 2 are ubiquitously expressed and calpain 3 is a skeletal muscle-specific homolog [79].

Calpain 1 and 2 target the thin filament protein complex, cleaving tropomyosin, tropomodulin, troponin T and I, and nebulin [73,80,81,82,83,84]. Calpain 1 and 2 do not degrade actin, suggesting that another factor involved in F-actin maintenance may also be required during thin filament turnover [84]. Calpain 1 and/or calpain 2 will release α-actinin while calpain 1 and/or calpain 3 cleave filamin C from the Z-disc [78,80,85,86]. All three calpains will free titin at the Z-disc, with most studies focused on calpain 3 and its localization to titin throughout muscle development [84,87,88,89].

Calpain activity can be modified by substrate phosphorylation. Phosphorylation of troponin I by cyclic AMP-dependent protein kinase (PKA) increases the resistance of troponin I to calpain 1 proteolysis [82]. However, troponin I phosphorylation by protein kinase C (PKC) significantly increases troponin I cleavage by calpain 1, while calpain 2-targeted degradation of troponin I was not altered by phosphorylation. Calpain 1-mediated cleavage of filamin C can be inhibited by phosphorylation using PKCα [78].

Calpain proteolysis is the first step in the turnover of several sarcomere proteins for protein quality control systems but calpain cleavage is not the endpoint for these proteins. Damaged proteins released from the myofiber are targeted and degraded by either the UPS or autophagy pathways.

### 3.3. Turnover of Thin Filament Components via E3 Ligases and the Ubiquitin Proteasome System

The UPS is dependent on three ubiquitin ligases, E1, E2, and E3, which recognize and deliver poly-ubiquitinated target proteins to the proteasome for degradation. E3 ubiquitin ligases (MuRF(s)) recognize damaged client proteins and transport them to E1 and E2 ligases for ubiquitination and subsequent degradation by the proteasome. For a recent detailed review of the UPS in muscle, see [75].

MuRF1 or MuRF2 recognizes nebulin, troponin T and I, and NRAP for UPS-mediated degradation (Figure 2B) [90,91,92]. Troponin T and I, tropomyosin, G-actin, myotilin, and α-actinin are all recognized and targeted for degradation by TRIM32 [93,94,95]. Despite the discovery of MuRF3 at the beginning of the century, filamin C and FHL2 are the only documented targets of MuRF3 [96,97]. A fourth muscle-specific ring finger protein has been discovered in the vertebrate lineage, excluding avian and placental mammals, but no targets of this MuRF4 have been identified yet [98].

Although MuRF factors work alone or in combination with another MuRF to target sarcomere proteins to the proteasome, other E3-ligases are composed of several proteins. The SCF (SKP1-CUL1-F-box protein) protein complex is composed of four proteins that recognize and target sarcomere components for degradation via the UPS system [99]. Fbxl22 is a cardiac-specific interchangeable recognition component of the SCF-E3 ligase complex that targets filamin C and α-actinin (Figure 2B) [99,100]. Fbxl22 can be replaced by atrogin-1, which localizes to the Z-disc and targets calcineurin for degradation, identifying a UPS therapeutic target for hypertrophy signaling [100,101,102]. Mutants of atrogin-1 show decreased troponin I degradation, suggesting troponin I might be an SCF-atrogin-1 target.

The co-chaperone bag3 and chaperone Hsc70 regulate UPS-mediated degradation of CapZ and indirectly F-actin stability [40,103]. Bag3 directs CapZ localization to the ends of F-actin anchored within the Z-disc. Bag3 association with CapZ recruits Hsc70 and binding of Hsc70 to the CapZβ1 subunit induces a conformational change in CapZ that stabilizes F-actin [42,104,105]. Without bag3, CapZ does not localize properly nor can it form stable bonds with F-actin, leading to degradation via the ubiquitin-proteasome system.

The 26S proteasome is the final destination for proteins targeted by the ubiquitin-proteasome system [75,106,107]. Although no calpain or E3 ligase that recognizes troponin C has been identified to date, troponin C is degraded by the 26S proteasome [80,106]. KLHL40 remains in the sarcomere after assembly to help stabilize nebulin and eventually leiomodin 3 [26]. KLHL40 protects nebulin and leiomodin 3 from targeted degradation by the ubiquitin-proteasome pathway.

### 3.4. Autophagy-Dependent Mechanisms for Thin Filament Maintenance

Autophagy is the second major degradation mechanism and differs from the UPS in that autophagy targets insoluble proteins, protein aggregates and lasting proteins [108]. The autophagy-lysosomal pathway (ALP) shares many similarities to the UPS system in that ALP uses E1, E2, and E3-like factors to expand an isolation membrane around the target organelle or insoluble proteins [109]; for a detailed review of this pathway, see [77,110]. Autophagy, like the UPS, is required for the maintenance of striated muscle due to the necessity of protein turnover and clearing of damaged sarcomere proteins to prevent protein toxicity [111,112,113].

Bag3, which was discussed in the UPS section, is also a member of an autophagy-dependent pathway called chaperone-assisted selective autophagy (CASA) [114,115]. Bag3 and SYNPO2 colocalize to the Z-disc and bind α-actinin and filamin C. Bag3 and SYNPO2 monitor filamin C integrity and recruit the CASA complex upon filamin C unfolding. The CASA complex is composed of Hsp70, Hsp27, HspB8, and the chaperone-associated ubiquitin ligase, CHIP [113,116,117]. Filamin C and SYNPO2 are removed from the sarcomere, ubiquitinated and targeted to the lysosome [116,118]. SYNPO2 is consumed in this pathway and would require continuous replacement as damaged/unfolded filamin C is turned over [115].

The autophagy-lysosomal pathway can also be triggered by the aggregation of actin protein. Actin aggregates also recruit and contain proteasomes (UPS) but the proteasomes are unable to degrade the actin aggregate, possibly due to the size or insolubility of the aggregate [116,119].

### 3.5. Dynamic Actin Turnover

Actin is the major component of the thin filament, interacting with all the dynamic proteins of sarcomere contraction. Therefore, it should be no surprise that actin must be consistently monitored and replaced due to normal wear and tear. This section describes actin protein turnover from CapZ’s weak dissociation to the re-polymerization of actin in the thin filament.

**Figure 3:**
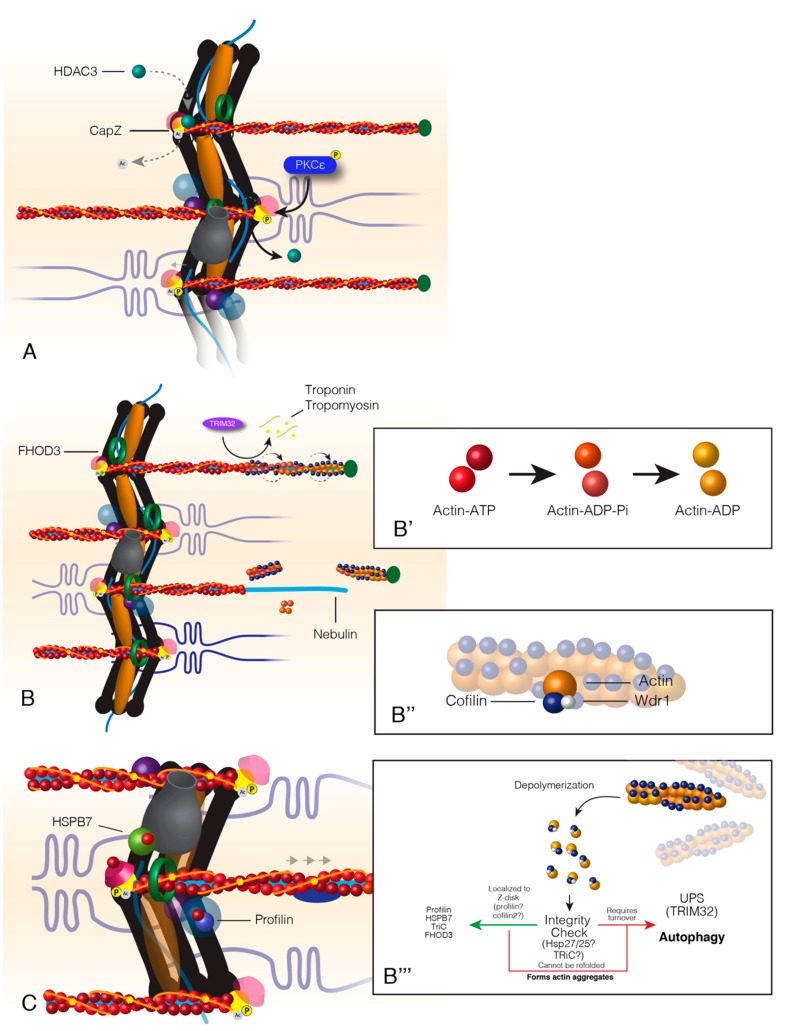
Turnover of actin within sarcomere thin filaments. Actin turnover begins with the relaxation of CapZ-thin filament binding at the Z-disc (**A**). This relaxation occurs from the competition between HDAC3 and PKCε. HDAC3 acetylates CapZ, which promotes a tight bond between CapZ and actin. PKCε phosphorylates CapZ that causes HDAC3 to disassociate from the Z-disc and CapZ to loosely bind the thin filaments. Following CapZ relaxation, FHOD3 encompasses the thin filaments, and actin-binding proteins associate with actin at the distal end of the thin filament (**B**). Actin at the distal end of the thin filaments is “aged” by the associated state of ATP, ADP+Pi or ADP (B’). Actin-binding proteins, Cofilin and Wdr1, bind “aged” actin, twist and break it away from the filament (**B**&**B’’**). Cofilin breaks filamentous actin into monomeric actin that is escorted back to the Z-disc where it is recycled back into the thin filaments (**B’’’**). Hypothetically, actin should pass through an “integrity check” as most sarcomere proteins do. The outcomes of this check would result in recycling back into the thin filament or targeted degradation (**B’’’**). Healthy actin monomers (G-actin) are reincorporated into the thin filament by Z-disc localized factors profilin, formin, and HSPB7; a process that maintains the oldest actin within the sarcomere A-band or the distal portion of the thin filament (**C**).

The binding between CapZ and actin is weakened by the phosphorylation of CapZ by PKCε, which causes HDAC3 to disassociate from CapZ (Figure 3A) [44,120]. HDAC3 disassociation allows for the acetylation of CapZ that then releases actin and allows for polymerization of the thin filament [41,44]. These posttranslational modifications of CapZ are reversible upon completion of thin filament turnover.

Cofilin2 is the only ADF (actin-depolymerizing factor)/cofilin protein family member present in muscle [121]. Cofilin2 is not required for sarcomere assembly as knockout mouse models develop muscle normally but quickly lose sarcomere integrity shortly after birth [122,123]. The cofilin co-factor Wdr1 (AIP-1) is required for cofilin-dependent actin depolymerization and disassembly of the thin filament (Figure 3B) [124]. Cofilin2 contains a region of surface residues that are hypothesized to act as a sensor of the nucleotide state of actin [125]. Cofilin2 preferentially binds actin-ADP at the pointed ends of the actin thin filaments by localization to the M-band of the sarcomere (Figure 3B, B’ & B’’). ADF factors regulate actin disassembly by changing the twist of actin filaments allowing for the severing of F-actin [122]. F-actin is further depolymerized into G-actin monomers to recycle actin and prevent aggregation of F-actin (Figure 3B’’’,C) [122,126,127,128,129].

Major sarcomere structural proteins (e.g., myosin, actinin) must be correctly folded and maintain structural integrity to be recycled back into the sarcomere during protein turnover. Protein recycling is possible if chaperones can correctly refold their client proteins but weakened or damaged targets that cannot reach their final conformation prevent reuse. In the event of a damaged/misfolded client protein, chaperones aid in the targeting of client proteins for degradation. Although no studies have identified an integrity check for actin recycling, it can be hypothesized that actin must also be correctly folded for re-incorporation into the thin filament (Figure 3B’’’,C). Unfolded/misfolded actin may recruit actin-binding partners due to exposed target residues. TRiC and/or Hsp27/25 are likely candidates for chaperones involved in a hypothetical actin integrity check due to their actin folding activity and localization to F-actin during muscle injury, respectively [61,130,131,132,133]. G-actin monomers recruit several binding partners, which supports a tight regulation of cytoplasmic concentrations of G-actin [61,125,134,135]. G-actin, when left “unattended” will polymerize into unstable/unregulated F-actin on its own [61,134,136,137].

After being severed from F-actin by cofilin2, ADP is exchanged for ATP in G-actin before being reincorporated into a developing thin filament [135,138]. While not definitively understood, profilin may facilitate this process by binding and forcing a conformational change in actin that favors the release of ADP [138,139,140]. Borrowing from the dendritic model for actin recycling at the leading edge of motile cells [59], profilin binds ADP-G-actin, undergoes nucleotide exchange to ATP-G-actin and is transferred from profilin to thymosin β4 (Tβ4). Tβ4 localizes G-actin to the barbed end of an actin thin filament at the Z-disc where G-actin is transferred to a separate pool of Z-disc localized profilin [141,142]. It is suggested that Tβ4 acts to create a separate pool of ATP-G-actin that both regulates thin filament polymerization and prevents uncontrolled F-actin synthesis and mouse Tβ4 mutants have short sarcomere thin filaments [59,141,142]. However, the use of Tβ4 to transfer G-actin from pointed to barbed ends of actin filaments seems inefficient since profilin is required at both ends for actin recycling.

A second model for actin recycling relies on reusing factors that were involved in sarcomere assembly. DAAM1/2, a formin protein, is localized to the Z-disc, where it recruits profilin and synthesizes F-actin during sarcomere assembly [48,51,55,143]. FHOD3, the debated formin discussed in thin filament assembly, is required for sarcomere maintenance by regulating actin dynamics and a lack of formin proteins increases the cytoplasmic concentration of G-actin [51,143,144]. FHOD3 likely accepts G-actin from profilin, but it is debated if this transfer occurs at the Z-disc or along the thin filament within the A-band [47,52,53,144,145,146].

Growing thin filaments will extend out from the Z-disc and are stabilized by leiomodin [71,147]. PKCα regulates thin filament length via phosphorylation of tropomodulin, which associate with the ends of actin filaments and stop polymerization [74]. How tropomyosin and troponin re-associate with the refreshed sarcomeric actin thin filament is unknown.

## 4. Diseases of Components of Striated Muscle Thin Filaments

This section will focus on diseases that arise solely from mutations that affect thin filament structural proteins or the factors that are required for proper assembly and maintenance. As some factors/pathways are shared with the development and health of other tissues (e.g., autophagy), the mutations discussed in this section will be specific to sarcomeres.

### 4.1. Mutations That Interfere with Proper Thin Filament Assembly

Mutations that disturb the proper assembly of sarcomere thin filaments can be further subdivided into several categories but in this review, we simplify the mutations to those that inhibit sarcomere formation and those mutations that develop weakened or improper thin filaments. Mutations that completely inhibit sarcomere formation are typically embryonic lethal due to the lack of a contractile heart and do not allow for a full pathophysiological study of these fatal mutations. The second category, which we will focus on in this review, encompasses those mutations that result in an initial weakly functional protein or misregulation of sarcomeric thin filament formation. Myopathies that manifest later in life from mutations in structural components should not be confused with maintenance or turnover myopathies as structural proteins fall under the umbrella of thin filament formation. Maintenance and turnover factors and their resulting myopathies will be discussed in the next section.

**Figure 4:**
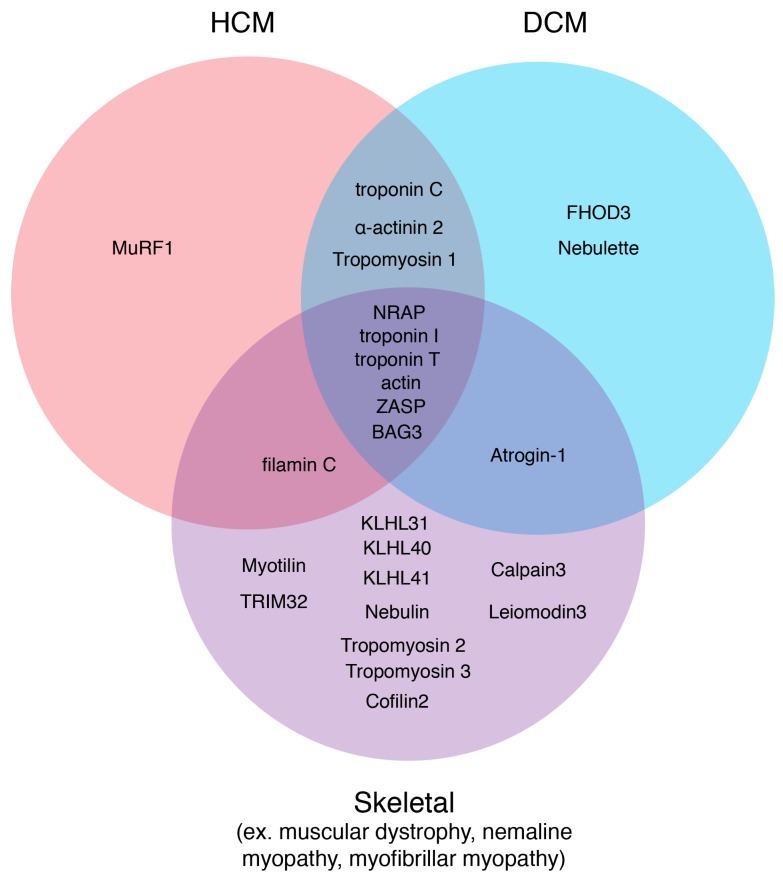
Sarcomere thin filament disease candidates. Using the online database, Online Mendelian Inheritance in Man, diseases originating from sarcomere thin filament factors were sorted into three disease states: HCM, DCM, and Skeletal. Factors that could carry mutations that could result in several different myopathies are depicted in the overlapping regions.

Weakly functional proteins are largely structural proteins of the sarcomere (e.g., actin, troponin T or I, tropomyosin, etc.) that do not maintain integrity over a period of time or for which the system can no longer compensate [148]. That time period can range such that myopathy presentations occur perinatally, pediatrically or with late onset. Improper functional leiomodin or tropomodulin result in irregular thin filament lengths that are either too short or too long; both inhibit efficient muscle contraction [149]. Mutations in many sarcomere structural proteins result in dilated cardiomyopathy (DCM) that can present early in childhood but many develop as late-onset DCM (Figure 4) [148]. Mutations in ZASP result in myopathies including muscular dystrophy, myofibrillar myopathy, DCM or HCM that manifest within and after the fourth decade [150,151,152]. NRAP mutations manifest as DCM in young adults [153], NRAP overexpression results in right ventricle cardiomyopathy in mice [154] and nemaline myopathy in zebrafish [24]. KLHL40 mutations have a perinatal presentation with severe paralysis to complete “lock-in” diagnoses due to a lack of nebulin and leiomodin stability [26]. Only a subset of thin filament proteins can carry DCM, HCM or nemaline-causing mutations that manifest at a variety of ages with a number of severities, suggesting that multiple disease mechanisms exist within muscle and possibly coordinate through these select factors (Figure 4) [148]. The mechanisms behind the age of onset are unknown but would be particularly powerful for therapeutic intervention.

### 4.2. Mutations that Interfere with Thin Filament Maintenance and Turnover

Maintenance and turnover of thin filaments refer specifically to those proteins/factors that were not required for the initial assembly of the sarcomeric thin filaments but are necessary to maintain thin filaments throughout the life of the organism. This category is composed mainly of accessory factors to the thin filaments and in rare cases, includes factors that are also necessary for thin filament formation (e.g., Kelch factors).

KLHL31 maintains muscle integrity by maintaining functional filamin C protein levels in the cell through ubiquitination [155]. KLHL31 knockout mice develop postnatal myopathies due to large FLNC aggregates that reduce Z-disc stability. TRIM32 mutations lead to variations of limb-girdle muscular dystrophy 2H and myofibrillar myopathy with accumulations of client protein aggregates (ex. myotilin) that interfere with normal muscle function [95]. Atrogin-1 mutations result in the misregulation of calcineurin and the autophagy pathway allowing for toxic protein accumulation within the cardiomyocytes that presents as pediatric DCM [101].

Bag3 is a unique factor that is involved in both the proper assembly and maintenance of the sarcomere [40,156]. Mutations in bag3 can result in embryonic lethality due to improper sarcomere assembly [157,158] or child-onset skeletal myopathies, DCM, or HCM (Figure 4) [159,160].

## 5. Summary

Although the mechanisms and development of sarcomere assembly have been, and are currently, explored, the focus of these investigations is primarily myosin, titin, and actinin. Only in the last decade has the importance of assembly and protein turnover studies of thin filament proteins been revealed due to a growing population with devastating mutations in these factors. Understanding the mechanisms behind sarcomere thin filament formation and maintenance is vital to creating a powerful and effective treatment for such myopathies.

## Figures and Tables

**Table 1:** Alternative names for sarcomere thin filament factors.

Common Name	Alternative Name(s)	Common Name	Alternative Name(s)
ZASP	Cypher, Oracle	HSPB7	Cardiovascular HSP
actinin	α-actinin	Muscle LIM Protein (MLP)	Cardiac LIM protein (CLP)
NRAP	N-RAP	T-CAP	TCAP, telethonin
Filamin C	γ-filamin, ABP-L, sarcomere filamin	Calpain 1	μ-calpain, mu-calpain, calpain I
myopodin	SYNPO2	Calpain 2	m-calpain, calpain II
Calsarcin	FATZ, myozenin	Calpain 3	p94
Calcineurin	Protein phosphatase 2B (PP2B)	MuRF1	TRIM63
KLHL41	Krp1, sarcosin	MuRF2	TRIM55
Titin	Connectin	MuRF3	TRIM54
CapZ	β-actinin, CapZβ, CapZβ1	Atrogin-1	MAFbx1, Fbxo32
Myotilin	TTID	Hsc70	HSPA8
TRiC	CCT, chaperonin	HspB8	Hsp22
Prefoldin	GimC	Cofilin2	m-cofilin, ADF
Hsp25/27	Hspβ1, Hsp25, Hsp27	Wdr-1	AIP-1
αβ-crystallin	CRYAB, Hsp20		
